# Mirror Therapy Reduces Pain and Preserves Corticomotor Excitability in Human Experimental Skeletal Muscle Pain

**DOI:** 10.3390/brainsci14030206

**Published:** 2024-02-23

**Authors:** Keita Nishi, Takefumi Moriuchi, Ryohei Okamura, Takashi Hasegawa, Xiaoqian Chang, Shinichi Matsumoto, Hironobu Koseki, Toshio Higashi

**Affiliations:** 1 Department of Physical Therapy, School of Health Science, Toyohashi Sozo University, 20-1 Matsushita, Ushikawa-cho, Toyohashi 440-8511, Japan; k-nishi@sozo.ac.jp; 2 Graduate School of Biomedical Sciences, Nagasaki University, 1-7-1 Sakamoto, Nagasaki 852-8520, Japan

**Keywords:** corticomotor excitability, experimental muscle pain, hypertonic saline, motor-evoked potentials, mirror therapy, transcranial magnetic stimulation

## Abstract

Approaches to preserve corticomotor excitability (CE) are attracting interest as a treatment for pain-induced changes in neural plasticity. We determined the effects of mirror therapy (MT) on skeletal muscle pain. Fifteen healthy adults who received hypertonic saline injections (5.8% NaCl, 0.2 mL) into the first dorsal interosseous (FDI) muscle of the right hand to induce experimental skeletal muscle pain were assigned to either the “MT and injection” or “injection only” group. Post-injection, the “MT and injection” group observed their left index finger abducting and adducting for 4 min, creating the illusion that the right index finger was moving. The “injection only” group remained at rest. CE and pain were assessed by measuring motor-evoked potentials (MEPs) of the right FDI triggered by transcranial magnetic stimulation and the numerical rating scale (NRS), respectively. MEP amplitudes were significantly higher in the “MT and injection” group, a trend that persisted post-MT intervention (MT intervention; *p* < 0.01, post-1; *p* < 0.05). The time for the NRS score to reach 0 was notably shorter in the “MT and injection” group (*p* < 0.05). Our preliminary results suggested that MT decreases CE and pain in skeletal muscles, potentially preventing neural plasticity changes associated with skeletal muscle pain and providing early pain relief.

## 1. Introduction

Acute pain can affect many aspects of motor control and performance, including changes in muscle strength, endurance, and force control [1,2,3]. Chronic pain also alters behavior and movement patterns, leading to pain avoidance [4,5,6]. These changes lead to long-term changes in corticomotor excitability (CE) that might cause plastic changes in the central nervous system [7,8]. This notion is supported by the experimental acute muscle pain decreases in the CE of painful muscles [9].

In studies where short-lasting pain is induced in healthy participants, it has been shown that a decrease in CE occurs. This decrease is thought to be a physiological defense mechanism, protecting the painful area from further pain or damage [9]. Conversely, in cases of persistent pain, it has been reported that the more severe the pain, the greater the decrease in CE [9]. In addition, since it has been reported that increasing CE using non-invasive brain stimulation techniques, such as high-frequency repetitive transcranial magnetic stimulation, alleviates pain in chronic pain patients, it is conceivable that a relationship exists between persistent pain and CE [10]. Therefore, if the attenuation in CE induced by pain can be restored before transitioning to chronic pain, it holds the potential to prevent the development of chronic pain. Furthermore, recent reports have highlighted that exercise can activate endogenous descending pain inhibitory pathways, a phenomenon being increasingly recognized as exercise-induced hypoalgesia [11].

Fractures, common orthopedic injuries necessitating immobilization for bone and joint recovery, have been reported to cause organic changes in both the peripheral and central nervous systems, inducing mechanical hyperalgesia and other symptoms [12,13]. Particularly, in the central nervous system, these changes have been observed at the cortical level, which may lead to further dysfunction and prolonged pain [14,15]. To remedy this situation, it is necessary to develop interventions that allow exercise to be performed without exerting physical stress on the affected region. Additionally, restoring the decrease in CE in the injured cortical areas is essential [16].

**Figure 1:**
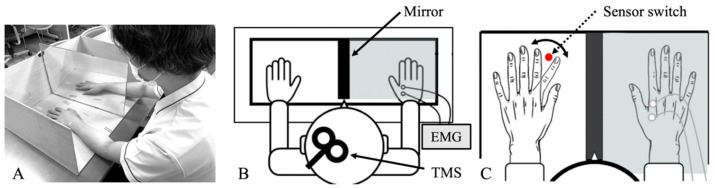
Mirror therapy task. (**A**) Equipment setup required for mirror therapy. (**B**) Motor-evoked potential measurements. Mirrors were concealed from the “injection only” group during measurements. (**C**) The mirror therapy task in the “MT and injection” group involved observation of abduction and adduction movements of left index finger. Transcranial magnetic stimulation was synchronized with abduction movement timing using infrared sensors to detect movement of left index finger. Sensor was positioned on the surface on which hands were placed. EMG, electromyography; MT, mirror therapy; TMS, transcranial magnetic stimulation.

Hypertonic saline is widely used to induce experimental pain, and CE can be assessed as motor-evoked potentials (MEPs) using transcranial magnetic stimulation (TMS). These techniques have been applied to reduce pain-induced CE [17,18,19,20]. The hypertonic saline-induced decrease in CE caused by pain in an experimental skeletal muscle pain model can be inhibited by action observation and motor imagery tasks that facilitate CE [21,22]. However, although active observation and motor imagery affect the nervous system, they do not alter the perception of pain itself [22]. Thus, we focused on mirror therapy (MT) as a potentially effective treatment modality (Figure 1A). Movement of an unaffected extremity is used to facilitate the CE involved in the movement of an affected extremity [23,24,25]. MT was originally used to treat phantom limb pain in amputees [26]. Pain reduction is thought to be caused by the illusion of extremity movement triggered by MT and facilitation of CE through the mirror neuron system by action observation [27]. MT can enhance excitability in the motor limb and ipsilateral motor cortex [28]. Furthermore, considering the effectiveness of physical therapy in mitigating pain [29], combining MT with actual exercise might reduce pain.

MT can activate the neural network involved in moving an injured extremity, even in the absence of physical movement [28]. Thus, it might be a useful treatment for acute musculoskeletal pain. We speculated that the visual illusion created by MT and its ability to facilitate CE might reduce pain.

This study aimed to determine the effects of MT tasks on pain intensity and CE in acute experimental skeletal muscle pain. We tested the hypotheses that MT counteracts decreases in CE associated with experimental muscle pain and reduces pain.

## 2. Materials and Methods

### 2.1. Participants

The inclusion criteria comprised being right-handed, being healthy, being aged ≥ 18 years, having no pain due to injury or other causes at the time of recruitment, and being without previous upper extremity pain or injury that required treatment from a healthcare professional. All participants were screened for the risk of being affected by TMS and injections and were given verbal and written explanations about the study before they provided written informed consent to participate. The study was approved by the local Ethics Committee of the Nagasaki University Graduate School of Biomedical and Health Sciences (approval no.: 19101004-3; date of approval: 8 March 2022). All the experimental procedures were conducted in accordance with the principles embodied in the Declaration of Helsinki.

### 2.2. Experimental Design

**Figure 2:**
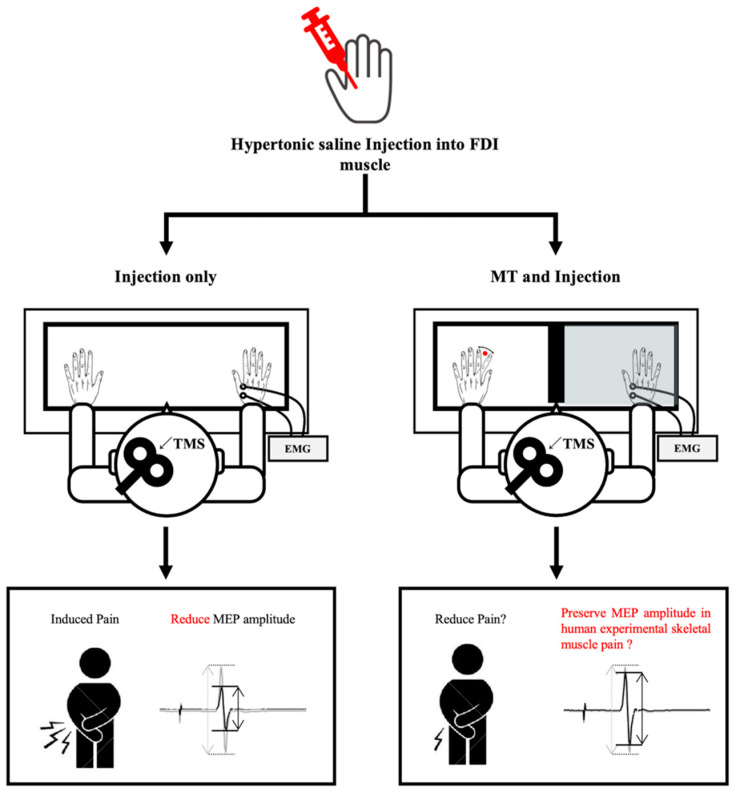
A schematic diagram of the design of the present study. EMG, electromyography; FDI, first dorsal interosseous muscle; MEP, motor-evoked potential; MT, mirror therapy; NRS, numerical rating scale.

The experimental design is shown in Figure 2. Participants were randomly assigned to the either the “injection only” or “MT and injection” group. In the “injection only” group, hypertonic saline was injected into the first dorsal interosseous (FDI) muscle, after which participants remained at rest without any movement while their pain status was monitored. Conversely, in the “MT and injection” group, the same hypertonic saline solution was injected into the FDI muscle. However, they performed the MT task for 4 min, starting from 60 s to 300 s post-injection, while their pain status was monitored.

**Figure 3:**
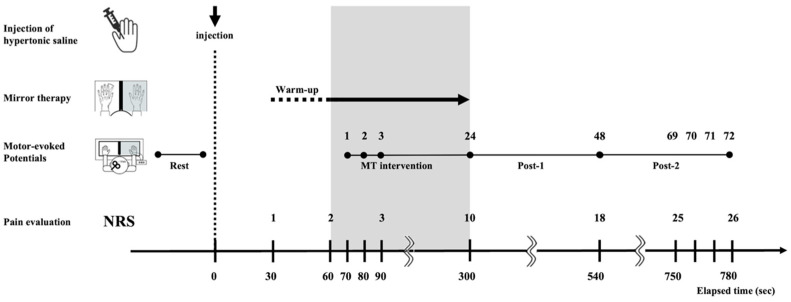
Experimental protocol. The “MT and injection” group was injected with hypertonic saline; then, the next 30–60 s were designated as the preparatory exercise period for MT. Abductive/adductive left index finger movements proceeded or were observed at 1 Hz rhythms from 60 to 300 s. Thereafter, participants rested with eyes closed for 780 s. Motor-evoked potential amplitudes were measured from 70 s after injection to 780 s at 10 s intervals (72 shots). MT, mirror therapy; NRS, numerical rating scale.

Baseline CE for both groups was assessed with 1 min of MT practice, followed by 3 min of rest before injection [30]. All participants were instructed to rest with their eyes closed, and then were evaluated with 20 MEPs obtained from random TMS pulses at 6–8 s intervals programmed using LabVIEW (National Instruments, Austin, TX, USA). All participants were exposed to TMS pulses every 10 s from 70 to 780 s after hypertonic saline injection, and 72 TMS MEPs per participant were recorded. We aligned MEPs during MT with the timing of left FDI contractions in the “MT and injection” group using an infrared sensor switch (KM221-010; Unique Medical Co., Ltd., Tokyo, Japan) and synchronously recorded TMS triggers using the LabVIEW system. The MEPs in the “injection only” group were recorded while the participants rested with their eyes closed (Figure 3).

### 2.3. Measurement of Corticospinal Excitability

Corticospinal excitability was assessed in each participant by recording the MEPs induced by TMS. The peak-to-peak MEP amplitude was measured over the FDI muscle in every trial. Analysis of the MEP amplitude was conducted using peak-to-peak values.

Surface electromyography (EMG) activity was recorded in the FDI muscles using pairs of 9 mm Ag–AgCl surface cup electrodes (SDC112, GE Healthcare; Chicago, IL, USA). Surface EMG signals were amplified and filtered at a bandwidth of 5–3000 Hz using a digital signal processor (Neuropack Sigma MEB-5504, Nihon Kohden; Tokyo, Japan). Analog outputs from a single processor were digitized at a sampling rate of 10 kHz and saved onto a computer for off-line analysis using an A/D converter (PowerLab16/30, AD Instruments; Bella Vista, NSW, Australia) (Figure 1B).

At the beginning of the experiment, we identified the optimal TMS coil position for evoking MEPs in the right FDI muscle (the hotspot). TMS was delivered to the left primary motor cortex hotspot, marked with a pen on a swimming cap covering the scalp of each participant. TMS employed a 70 mm figure-of-eight coil connected to a magnetic stimulator (Magstim 200, Magstim, UK). The coil was positioned tangentially over the scalp with its handle pointing backward and rotated approximately 45° away from the mid-sagittal line. Care was taken to maintain the same coil position relative to the scalp throughout the experiment. The resting motor threshold was defined as the lowest stimulus intensity required to evoke an MEP of at least 50 μV in amplitude in the right FDI muscle in 5 out of 10 trials. The test stimulus intensity was carefully adjusted to elicit a peak-to-peak MEP amplitude of approximately 1 mV in the FDI muscles under the control condition (110–130% of the resting motor threshold). Throughout the experiments, participants were instructed to avoid inadvertent movements that could give rise to background EMG activity. For each muscle in each trial, the 20 ms period preceding TMS initiation was checked for background EMG activity. If background EMG data were found, data from both muscles in the trial were rejected.

### 2.4. Mirror Therapy Task

The “MT and injection” group watched their left hand in a mirror during repetitive internal/external rotation movements of the left index finger at a rhythm of 1 Hz. Verbal encouragement was provided to facilitate the perception that their right hand was moving. All participants repeated the same MT task for 1 min before being injected with hypertonic saline. The “MT and injection” group then implemented the MT task for 4 min from 1 to 5 min thereafter. The first 30–60 s after injection were designated as a preparatory period that allowed the participants to synchronize their movements to the prescribed rhythm (Figure 1C).

### 2.5. Hypertonic Saline Injection and Measurement of Pain Intensity

The injection site was determined by palpating the contracted FDI muscle. The skin was disinfected with alcohol, and pain was induced by injecting a bolus of sterile hypertonic saline (0.2 mL, 5.8% NaCl) into the FDI muscle using a 1 mL syringe with a disposable 27 G needle [13,16]. Pain intensity was scored from 0 to 10 using a numeric rating scale (NRS) at 30 s after injection and every 30 s thereafter until the first NRS score of 0 indicated the disappearance of pain.

### 2.6. Statistical Analyses

We compared MEPs at rest, and at 0–300 (during MT intervention), 310–540 (post-1), and 550–780 (post-2) safter injection. We determined whether interactions or main effects occurred by evaluating MEP amplitude using two-way repeated measures analysis of variance (ANOVA) with both “groups” (“MT and injection”/“injection only”) and elapsed “time” after hypertonic saline injection as the main factors. A post-hoc test was conducted with Bonferroni adjustment to compare the “group” and with Dunnett’s test to compare the “time”.

We also compared the time elapsed between hypertonic saline infusion and pain resolution, defined as an NRS score of 0, between the groups using independent t-tests. In addition, we determined potential interactions or main effects by evaluating NRS scores using a two-way repeated measures analysis of variance (ANOVA), with both groups (“MT and injection” and “injection only”) and time elapsed after hypertonic saline injection as the main factors. The NRS scores in both groups were compared every 120 s from 30 s to 750 s (seven times in total) after the hypertonic saline was injected.

All data were analyzed using SPSS 23 (IBM Corp., Armonk, NY, USA). Statistical significance was set at <5%.

## 3. Results

### 3.1. Study Participants

Table 1 summarizes the characteristics of the “MT and injection” and “injection only” groups. One participant withdrew from the study due to developing vagal reflexes after hypertonic saline injection. Consequently, data from 15 participants were analyzed. Age, sex, and baseline MEP values did not significantly differ between the groups.

### 3.2. Response of TMS MEPs to Pain Induced by Hypertonic Saline

**Figure 4:**
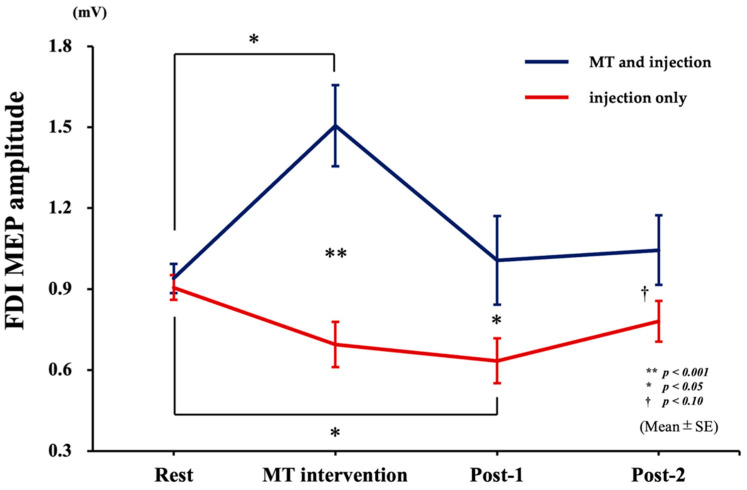
Motor-evoked potentials in first dorsal interosseous muscle. Data are shown as mean (±SE) FDI MEPs. Blue and red lines indicate “MT and injection” and “injection only” groups, respectively. Mirror therapy intervention and post-1 significantly differed between groups (* *p* < 0.05; ** *p* < 0.001). FDI, first dorsal interosseous muscle; MEP, motor-evoked potential; MT, mirror therapy; SE, standard error.

A two-way repeated measures ANOVA revealed a significant interaction between “group” and “time” (F_3,39_ = 5.531, *p* < 0.01, parietal *η*^2^ = 0.298). Post-hoc analysis with Bonferroni correction revealed significantly higher MEP amplitude in the “MT and injection” group than in the “injection only” group during MT intervention (*p* < 0.001), post-1 (*p* < 0.05), and revealed marginally non-significantly higher MEP amplitude in post-2 (*p* = 0.055). Post-hoc analysis with Dunnett’s test revealed significantly higher MEP amplitude in MT intervention compared to rest in the “MT and injection” group (*p* < 0.05) and significantly lower MEP amplitude in post-1 compared to rest in the “injection only” group (*p* < 0.05) (Figure 4).

### 3.3. Response of NRS Score to Pain Induced by Hypertonic Saline

We defined an NRS score of 0 as the time when pain disappeared. The independent t-test revealed that pain dissipated significantly faster in the “MT and injection” group than in the “injection only” group (*p* < 0.05; Table 2).

### 3.4. Time Course of NRS Scores

**Figure 5:**
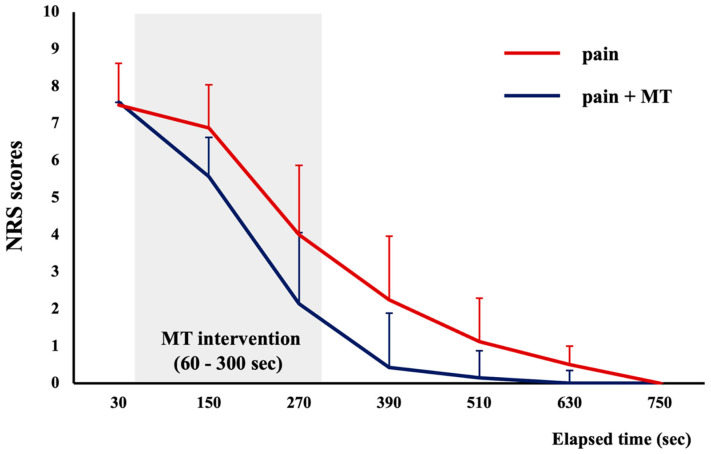
Numerical rating scale scores and timing of pain disappearance. Data are shown as means (±SDs). Blue and red lines indicate “MT and injection” and “injection only” groups, respectively. MT, mirror therapy; NRS, numerical rating scale.

The time courses of NRS scores for pain induced by hypertonic saline to pain dissipation are shown in Figure 5. A two-way repeated measures ANOVA revealed a significant main effect of “group” (F_1,13_ = 5.188, *p* < 0.05, parietal *η*^2^ = 0.285) and “time” (F_6,78_ = 128.697, *p* < 0.001, parietal *η*^2^ = 0.908). However, there was no significant interaction between “group” and “time” (F_2.9,37.8_ = 2.237, *p* = 0.102, parietal *η*^2^ = 0.147).

## 4. Discussion

Approaches that can preserve CE have recently attracted increasing interest owing to their potential application to musculoskeletal pain rehabilitation [16]. As far as we can ascertain, this is the first investigation into the impact of a visual illusion of hand movement on experimental skeletal muscle pain and associated neuroplastic changes. We applied a paradigm based on the mechanisms through which MT induces the visual illusion of hand movement and facilitates CE ipsilateral to the hand that is doing an exercise task. The results indicated that pain intensity and duration were significantly improved in the “MT and injection” group that applied the MT task during acute experimental muscle pain compared with the “injection only” group that did not. A decrease in CE associated with experimental pain was alleviated in the “MT and injection”, but not in the “injection only” group. These findings provide useful clinical insights and suggest that MT can reduce pain and restore CE in patients with acute musculoskeletal pain.

### 4.1. Acute Experimental Pain Reduces CE

We confirmed that CE manifesting as MEP amplitude in the “injection only” group after hypertonic saline injection was reduced; this agreed with previous findings [18,20,22]. The reduction in CE associated with pain is thought to be mediated via an increase in gamma-aminobutyric acid inhibition and a decrease in glutamate-mediated (N-methyl-d-aspartate receptor acting on glutamatergic interneurons) intracortical mechanisms [20]. The reduction in CE might serve as a protective mechanism against further injury by inhibiting the movement of an affected extremity [31,32]. Our results also confirmed that CE in the “injection only” group decreased soon after the hypertonic saline was injected and persisted throughout the 13 min monitoring period. A review of experimental studies of pain in healthy persons found that the decrease in CE was sustained for up to 30 min after pain resolution, and then returned to baseline levels [9]. Therefore, the decrease in CE identified herein during the monitoring period might be associated with the impact of experimental skeletal muscle pain.

### 4.2. Mirror Therapy Might Preserve CE in Human Experimental Skeletal Muscle Pain

We revealed that MT can preserve CE in human experimental skeletal muscle pain, possibly through its effects on the nervous system.

MT can facilitate CE ipsilaterally to the hand being exercised [28,33,34]. The primary effect herein was a change in the CE of the hand ipsilateral to that engaged in the exercise task caused by the visual illusion phenomenon. Unilateral hand movements are typically generated via excitation of the contralateral corticospinal tract, while the activity of the ipsilateral corticospinal tract is repressed (interhemispheric inhibition) [35]. However, when MT induces a visual illusion, it can enhance CE in the motor-related area of the hand behind the mirror that is not involved in the movement [28]. Thus, MT might have augmented CE of the motor-related area of the afflicted hand in the “MT and injection” group. The secondary effect was motor-related CE changes due to action observation that facilitates the CE involved in the observed movement (mirror neuron system) [29]. Here, the “MT and injection” group watched the exercising hand in the mirror, which might have facilitated CE in the movement-related areas of the hand with pain on the other side of the mirror. Furthermore, the “MT and injection” group was intentionally encouraged to create a visual illusion of hand motion by observing mirror images during MT. This might have induced or enhanced the induction of motor imagery during motor observation and enhanced the facilitating effect MT had on CE [25,30].

We found that the CE of the FDI, reflected as MEP amplitude, significantly increased in the “MT and injection” group compared with that in the “injection only” group, even after intervention. Studies of brain activity before and after MT have found increased CE on the same side as the hand that exercised even after treatment [28,36,37,38,39,40]. A study of a combined exercise observation and motor imagery intervention for experimental skeletal muscle pain found a significant increase in CE during and immediately after intervention compared with a group without intervention [22]. These findings suggest that the MT applied to experimental skeletal muscle pain in the present study counteracted the pain-induced decrease in CE and that this effect persisted after the intervention.

### 4.3. Mirror Therapy Reduced and Promoted Recovery from Acute Skeletal Muscle Pain

The results of this study confirmed that pain intensity and time to pain resolution in the “MT and injection” group was reduced compared with those in the “injection only” group, suggesting that MT could reduce experimental skeletal muscle pain. Pain intensity is rated lower during working memory tasks such as attention, Stroop, and three-back tasks. This decrease in pain intensity during task progression is thought to be mediated by a decrease in activity in pain-related brain regions as attention is diverted to the task [22,41]. The MT task in this study required participants to accomplish certain index finger movements, rhythms, and eye gazes. Therefore, their attention might have been diverted toward the task rather than pain during the MT intervention.

We consider that the MT task in this study was a painless hand exercise that might reduce pain and promote recovery, as exercise decreases sensitivity to pain stimuli [29]. This phenomenon is called exercise-induced hypoalgesia and has recently achieved popularity as a concept that might be effective for treating patients with intractable chronic pain [42,43,44]. Most exercises related to exercise-induced hypoalgesia, such as aerobic exercise and resistance training, require some degree of exercise load; therefore, the ability of the index finger exercise used in the MT task in this study to induce hypoalgesia must be verified.

The kinesthetic illusion effect might be the mechanism through which MT reduces pain. Kinesthetic illusions induced by MT are thought to enhance spatial attention toward an invisible affected limb [45]. MT stimulates activity in the primary visual and somatosensory cortices ipsilateral to a moving hand, as well as in higher-order processing areas in the occipital and parietal lobes [46,47,48,49,50,51]. In other words, inducing the kinesthetic illusion using a mirror image of a painful moving hand might activate neural networks associated with movement, including vision and perception. Increased attention to the painful hand might have reduced pain by creating a positive image of it moving despite the pain [52].

Because we accumulated CE data for up to 13 min after pain onset, we could not determine CE trends thereafter. Since CE decreased for ~30 min after hypertonic saline injection [9], the long-term effects of MT require further investigation. In addition, the effects of MT on patients with acute traumatic injuries, such as fractures, should be clinically investigated using randomized controlled trials before our results can be clinically applied. The advantages of MT are that it does not require hand movements on the injured side and carries little risk. Our findings indicate that understanding the effects of MT in clinical practice would be valuable.

Our study has some limitations. First, the sample size was small. The sample size calculation based on effect size required more than 40 participants. It is crucial to recognize that the findings of this study should be regarded as preliminary because data were obtained from only 15 participants. In the future, the number of participants should be increased, and the data should be augmented. Second, the conditions under which the control group was set were limited, and the effects of MT cannot be fully verified. Especially crucial is the establishment of appropriate controls for the “injection only” group to thoroughly scrutinize the outcomes of this study. Further verification in the future is necessary. Specifically, potential control groups could involve those solely engaged in action observation or motor imagery, individuals performing a working memory task, and participants executing the index finger exercise without a mirror display. We believe that future validation of these additional conditions will help elucidate the details of the effects of MT on skeletal muscle pain.

The clinical significance of this study is that MT has the potential to preserve CE at the site of injury that has been reduced by pain, thereby reducing the duration of pain. This may help prevent the chronicity of pain associated with immobility that occurs during periods of rest and immobilization necessary for the recovery of damaged tissue, a common requirement in many orthopedic conditions. In addition, one of the greatest advantages of MT is its ability to activate the neural networks associated with movement in the affected area in the absence of any physical stress on the area. This means that active therapeutic intervention is possible even during the period of immobilization of the affected area, which may contribute significantly to the acute treatment of musculoskeletal trauma.

## 5. Conclusions

Mirror therapy for experimental acute skeletal muscle pain could be an effective tool for preventing plastic changes in the central nervous system associated with pain. Our results concur with those of previous studies of mental practice. Furthermore, to the best of our knowledge, this is the first study to test an approach aimed at preserving the reduction in CE, which is effective against pain. Mirror therapy could be easily introduced into clinical practice because it requires simple tools, acts on the nervous system without the need to exercise the affected limb, and could be applied to any trauma or disease that causes pain in the musculoskeletal system. Our findings may give hope for many patients with pain that is difficult to treat.

## Figures and Tables

**Table 1:** Characteristics of the participants.

Characteristic	“MT and Injection”	“Injection Only”
Male/female (n)	4/3	4/4
Age (years)	24.9 ± 6.3	21.8 ± 2.4
Baseline MEP amplitude (mV)	0.94 ± 0.14	0.89 ± 0.12

MEP, motor-evoked potential; MT, mirror therapy; mV, millivolts.

**Table 2:** Comparison of the time when pain disappeared between groups.

	“MT and Injection”	“Injection Only”	*p*-Value
Time (ms)	394.3 ± 155.8	573.8 ± 155.8	*p* < 0.05

MT, mirror therapy; ms, milliseconds.

## Data Availability

The data that support the findings of this study are available from the corresponding author upon reasonable request. The data are not publicly available due to privacy restrictions.
